# The low level of plastome differentiation observed in some lineages of Poales hinders molecular species identification

**DOI:** 10.3389/fpls.2023.1275377

**Published:** 2023-12-08

**Authors:** Katarzyna Krawczyk, Łukasz Paukszto, Mateusz Maździarz, Jakub Sawicki

**Affiliations:** Department of Botany and Nature Protection, University of Warmia and Mazury in Olsztyn, Olsztyn, Poland

**Keywords:** chloroplast genome, super-barcoding, Poaceae, Poales, species identification, plastome variability

## Abstract

Chloroplast genomes are a source of information successfully used in various fields of plant genetics, including molecular species identification. However, recent studies indicate an extremely low level of interspecific variability in the plastomes of some taxonomic groups of plants, including the genus *Stipa* L., which is a representative of the grass family. In this study we aimed to analyze the level of chloroplast genome diversity within particular genera as well as the effectiveness of identifying plant species in the Poaceae family and the other representatives of Poales order. Analysis of complete plastid genome alignments created for 96 genera comprising 793 species and 1707 specimens obtained from the GenBank database allowed defining and categorizing molecular diagnostic characters distinguishing the analyzed species from the other representatives of the genus. The results also demonstrate which species do not have any species-specific mutations, thereby they cannot be identified on the basis of differences between the complete chloroplast genomes. Our research showed a huge diversity of the analyzed species in terms of the number of molecular diagnostic characters and indicated which genera pose a particular challenge in terms of molecular species identification. The results show that a very low level of genetic diversity between plastomes is not uncommon in Poales. This is the first extensive research on super-barcoding that tests this method on a large data set and illustrates its effectiveness against the background of phylogenetic relationships.

## 1 Introduction

Plastid genomes are widely employed in plant evolutionary research, encompassing areas such as phylogenetics, taxonomic implications, population genetics, and phylogeography (Huang et al., 2019) (Abdullah et al., 2021). The reason for which plastomes are willingly used in this applications are the same that predestinate it to be applied in molecular identification of species, i.e. mostly uniparental inheritance, relatively high level of polymorphism, and presence in multiple copies in a cell, which is of particular importance in the analysis of material with limited availability and herbaceous material (Palmer, 1985; Henriquez et al., 2014; Ramsey and Mandel, 2019). The method of species identification based on variability of nucleotide sequences, called DNA barcoding, has been developing dynamically since 2003 (Hebert et al., 2003) and in the case of its application in plants, from the very beginning (Kress et al., 2005) the main subject of research as a source of potential barcode sequences was the chloroplast genome. Looking for the most effective, universal and at the same time easy to amplify barcode sequence, the variability of many coding and non-coding candidate barcodes within the plastome was analyzed over the years. Initially, loci widely used in phylogenetic implications were tested for their usefulness in distinguishing representatives of individual plant species, among others: *trn*H-*psb*A (Kress et al., 2005), *trn*L (Taberlet et al., 2007), *atp*F-*atp*H, *mat*K, *rbc*L, *rpo*B, *rpo*C1, *psb*K–*psb*I (CBOL Plant Working Group1 et al., 2009), *atp*F-*atp*H (Lahaye et al., 2008), *trn*L-*trn*F (Ferri et al., 2015), *acc*D, *ndh*J (Ford et al., 2009). In the following years of research, the utility of numerous regions within the plastome was investigated, also including its fragment-by-fragment variability, regardless of function (Xu et al., 2017; Krawczyk et al., 2018; Jiao et al., 2019; Ślipiko et al., 2020; Sawicki et al., 2021).

Numerous studies have shown that unfortunately none of candidate sequences meet the requirements of the universal barcode for plants, therefore a research trend focused around searching for the best combination of two or more single loci in the so-called multi-locus barcodes (Hollingsworth et al., 2011; Li et al., 2015). Currently, the gold standard in barcoding, the core-barcode with the highest discriminatory power, recommended by the CBOL Plant Working Group (CBOL Plant Working Group1 et al., 2009) is the *mat*K-*rbc*L combination, which length in *Arabidopsis thaliana* oscillates around 1440 bp (Hollingsworth et al., 2011). Again, it is not ideal, mainly due to the low PCR efficiency of *mat*K amplification in some taxonomic groups, especially gymnosperms and cryptogams (CBOL Plant Working Group1 et al., 2009). In the absence of a universal, highly effective barcode for all plants, some researchers have turned to developing specific barcodes, designed for lower taxonomic groups - such as families or even genera, forgoing the universality of the method in favor of its effectiveness (Saddhe and Kumar, 2017; Zhang et al., 2021). However, the most prospective research trend that is currently developing seems to be the super-barcoding (Parks et al., 2009), the method which analyzes the variability of complete plastid genomes. Thanks to the huge development of high-throughput sequencing methods in recent years, and also the reduction of their cost, the analysis of whole-plastome variability is increasingly used in various studies, also including molecular identification of closely related land plant species (Kress, 2017; Fu et al., 2019; Chen et al., 2020; Li et al., 2021; Zhang et al., 2021; Guo et al., 2022) or even varieties within species (Ji et al., 2020; Jiang et al., 2023). The application of super-barcodes helps to increase efficiency of species identification and at the same time circumvents possible issues with low PCR efficiency (Li et al., 2015; Jiang et al., 2023).

The analysis of the complete plastome instead of standard barcodes, which individual length does not exceed 1 kb, may seem a little bit like using a sledgehammer to cut a nut, nevertheless, this approach is fully justified, as despite the high discriminative power of super-barcodes, in the case of some recently divergent species even they are not 100% effective. An example is the research on *Fritillaria* (Liliaceae) where super-barcode provided discriminatory power at the level of 87.5% (Wu et al., 2021). Much lower effectiveness of super-barcoding was reported for the Lauraceae family, where the use of the whole plastome sequence increased the efficiency of species identification only to 60% while the discrimination success obtained with the standard plant DNA barcodes was 40-50% (Liu et al., 2021). Another taxonomic group, which is a challenge in terms of both classical and molecular identification of species, due to high variability of features within genera, is the family of grasses (Poaceae) (Hand et al., 2013; Qiu et al., 2019). Studying the genetic variability of feather grasses (*Stipa* L. Poaceae), a strikingly low level of variability in plastome sequences between species pairs amounting from 0% to 0.3% (mean 0.01%) was observed (Krawczyk et al., 2018; Krawczyk et al., 2022). Confronting this result with significantly higher mean nucleotide variability values observed in *Taxillus* (Loranthaceae) (1.3%), *Dalbergia* (Fabaceae) (0.86%) or *Populus* (Salicaceae) (0.36%) plastomes (Su et al., 2021), the question arises whether the low level of differentiation observed in *Stipa* is a common phenomenon within grasses or whether it is exceptional in this taxonomic group. Despite the great sequence similarity of plastome genomes in feather grasses, the identification success in the genus reached 94.74% of species analyzed in the study (Krawczyk et al., 2018). This observation raises another question, whether low genetic variability translates into low success of molecular species identification. These questions seem to be relevant in the context of evolutionary genomics itself, but also from the point of view of the molecular species identification method. As in recent years apart from the methods based on the phylogenetic tree or genetic distance in barcoding studies, the development of methods based on molecular diagnostic characters (MDCs) (Merckelbach and Borges, 2020) can be observed, the need arises to study the effectiveness of this tool in different taxonomic groups so that in the future it can be determined what number of MDCs may be regarded a success of species identification.

To analyze the effectiveness of the super-barcoding method on a wide set of data, as well as taking into account that the essence of the barcoding method is the use of sequence resources available in public databases, data available in the GenBank database (Sayers et al., 2022) were used in the study.

This study aims to examine and compare the level of chloroplast genome diversity within particular genera as well as the effectiveness of identifying plant species in the Poaceae family against the background of the Poales order in relation to their phylogenetic relationships. The significant advantage of the availability of data on the chloroplast sequences of grasses over other families from Poales is the result of the emphasis placed in research on plants from the Poaceae family, due to the fact that grasses are important in the food, fodder, bioenergy, construction and horticultural industries, are the main component of meadows and bamboo forests, therefore are of great economic and ecosystem importance (Hodkinson et al., 2007; Soreng et al., 2017).

Recently published studies on the evolution of Pooideae indicated that one of the most important factors influencing the evolution of this group was the transition from closed to open habitats, that happened at the turn of Eocene and Oligocene. This event was associated with significant upshift of diversification rate in three Pooideae sublineages: core Pooideae, supertribe Stipodeae and supertribe Nardodae (Zhang et al., 2022). Moreover, the results of research showed that the occupation of open habitat might have subsequently facilitated the radiation and expansion of Pooideae. Taking into account the impact of the change of the habitat on the diversification of this taxonomic group, we also decided to analyze whether the currently occupied habitat type by species from the studied genera from Poales is related to the currently observed level of genetic diversity within the genus.

## 2 Materials and methods

### 2.1 Sequence acquisition and alignment

The data used in this research comes entirely from the GenBank database (Sayers et al., 2022), therefore the data collection is conditioned by the availability of complete chloroplast genome sequences in this public, open access database. The sequences were downloaded from GenBank in February 2022, and their accession numbers are given in **Supplementary Table 1**. In total, 1707 chloroplast genomes from 793 species representing 96 genera from Poales order were analyzed in the study. The vast majority of them, i.e. 90 genera, represent the Poaceae family, while Bromeliaceae and Cyperaceae are represented by two genera and Eriocaulaceae and Typhaceae only by one genus, respectively. Only those genera for which data on complete plastomes were available for at least three species were included in the analysis, hence the smallest number of species in the genus in the studied data set is three. The richest genus in terms of the number of analyzed species was *Andropogon*, for which plastome sequences were available for as many as 41 species. The analyzed data set is also very diverse in terms of the number of examined individuals per species ranging from one to 115 (*Brachypodium distachyon*). In the case of genera represented by very numerous sequences (*Oryza rufipogon* and *O. sativa*), the dataset used for further analysis was limited to 50 sequences due to the computational capabilities of the software used. Before comparative analyses, the IR regions for all the plastome sequences were identified and removed by IRScope (Amiryousefi et al., 2018) and seqinr R package (Charif and Lobry, 2007). The MAFFT (Katoh and Standley, 2013) software with default parameters was used to create multiple sequence alignment (MSA) for each genus. For each of the alignments created for the genus, mean pairwise sequence identity expressed as a percentage and representing the proportion of identical residues in the aligned sequence pairs was calculated. From each MSA for a genus consensus sequence was generated. The consensus sequences of each dataset were collected and realigned to obtain the final MSA for Poales. The both types of MSAs were used for downstream analyses, the genera MSA for MDCs identification and final MSA for phylogeny reconstruction. The phylogenetic Poales tree was built using the Maximum Likelihood (ML) method implemented in the IQ-TREE 2 (Minh et al., 2020). The *Sparganium* consensus sequence was selected as a root of the tree.

### 2.2 The analysis of molecular diagnostic characters

The MDCs analysis was performed using the FastaChar v. 0.2.4 software (Merckelbach and Borges, 2020). The analysis was performed by comparing each species with the other members of the same genus included in the study. As a result, a list of diagnostic features of individual species was obtained, generated in the form of spreadsheets. The FastaChar enables analysis of molecular diagnostic characters (MDCs) defined as so-called pure diagnostic sites (Sarkar et al., 2008) which are polymorphic sites (commonly SNPs) in the nucleotide alignment for which all the members of the query taxon have a given variant that is not present in any member in the reference taxa. Sites which are polymorphic within the query taxon were not included in the analysis. The number of MDCs for each of the analyzed species was compiled and the average number of MDCs for the genus was calculated (**Supplementary Table 2**). This value was also calculated in relation to the length of the alignment.

MDCs obtained from FastaChar software were evaluated by custom R script. In the first part of the script, the algorithm focused on converting xlsx results to data frame format and combining data from each genus to one matrix. After the preparation step, the script assumes identification and classification of MDCs to different types of variations. The main criteria of MDCs classification were length of consecutive nucleotides within the MDCs event and type of substitution or indel. According to that assumption the data was divided into deletion, insertion, single mixed variation, multi substitution, multi deletion, multi insertion and multi mixed variation. MDCs with more than one continuous nucleotide were characterized as multi variations. The mixed type was described in case of occurrence substitution and indel within a single MCDs event in the genera.

The minimum, maximum and mean of MDCs, family membership, habitats and percentage of identity within genera were visualized on circular plots created by R bioconductor packages, e.g. ggtreeExtra (Xu et al., 2021), phyloseq (Revell, 2012), circlize (Gu et al., 2014), ggtree (Yu et al., 2017).

### 2.3 Statistical analyses

Considering the differentiated number of species analyzed within individual genera, we checked whether there was a relationship between the number of species studied in the genus and the sum of MDCs in a genus. It was also tested whether the obtained results were significantly biased by the number of individuals analyzed within each species. The distributions of the studied dependent variables were tested for compliance with the normal distribution using the Shapiro-Wilk test. In all cases (sum of MDCs in a genus; MDCs per alignment length x 10-4; average number of MDCs in a genus), the distributions of the result variables were significantly deviated from the normal distribution (Shapiro-Wilk test, p < 0.00001) and positively skewed (skewness > 2.14). Therefore, in the analysis of the relationship between the studied variables and the predictors, generalized linear models (GLM) were used, allowing for testing the relationships between the predictors and the outcome variable. For the result variables (sum of MDCs in a genus; MDCs per alignment length, average number of MDCs in a genus) belonging to the count type, the Poisson distribution was adopted. Data describing the number of species in the genus, the number of individuals in the genus and mean pairwise sequence identity within a genus were logarithmic scaled. Statistics were calculated according to the algorithms of the Statistica 13.3 package.

The representativeness of the analyzed data was also examined by verifying what percentage of species distinguished within a given genus covers the data set we analyzed. When determining the number of species belonging to each genus, the list of species on the World Flora Online database (World Flora Online, 2023) was used.

### 2.4 MDCs in relation to the habitat type

Each genus analyzed in the study has been characterized in terms of the dominant type of habitats occupied by the representing species. The genera were assigned to one of three habitat categories: open, woodland or mixed basing on the available databases and publications (Florabase, 1998; AusGrass2, 2014; Grass Base - The Online World Grass Flora, 2014; Useful Tropical Plants Database, 2014; Nowak et al., 2020; Coastal Plain Plants, 2021; Lady Bird Johnson Wildflower Center, 2023; Native Plant Trust, 2023; The PLANTS Database et al., 2023; World Flora Online, 2023). The open habitats category includes grasslands, sand dunes, fields, wetlands, grassy hillsides and mountain slopes. Species occurring in the forest undergrowth as well as plants forming bamboo forests were classified to the woodland category. The mixed category includes species occurring in forest margins, savannas, thickets, ruderal habitats and species growing in both woodland and open areas.

The comparison of the mean pairwise sequence identity [%] calculated for each genus with the distinguished type of environment was tested with the Kruskal-Wallis test, and the differences between pairs of studied variables were tested with Dunn’s multiple comparisons test.

## 3 Results

### 3.1 MDC against the background of the phylogenetic tree

The length of the 96 analyzed sequence alignments created for each particular genus ranged from 113,256 bp in *Eremopyrum* to 144,526 bp in *Carex* (**Supplementary Table 2**). Phylogenetic reconstruction based on consensus sequences generated from these alignments resulted in a well resolved phylogenetic tree (**Supplementary Figure 1**), with family Poaceae clearly divided into two sister clades, one comprising species from Bambusoideae, Oryzoideae and Pooideae subfamily (BOP clade) and the other one comprising representatives of Panicoideae, Aristidoideae, Chloridoideae, Micrairoideae, Arundinoideae and Danthonioideae (PACMAD clade), while the inner clades comprising individual subfamilies were highly supported.

The number of MDCs that distinguish a species from the other representatives of the same genus was extremely variable ranging from zero in numerous species to 8,672 in *Carex siderosticta*. The *Carex* genus was also characterized by the highest sum of MDCs calculated for the whole genus reaching 32,195. An average number of MDCs in a genus ranged from zero in *Secale* to 3,071 in *Eriocaulon*. (**Supplementary Table 2**).

**Figure 1:**
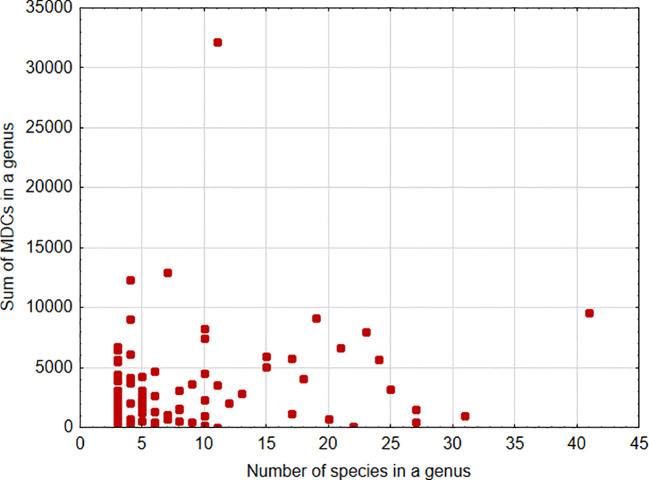
Sum of MDCs calculated for the genus versus number of species analyzed within the genus.

**Figure 2:**
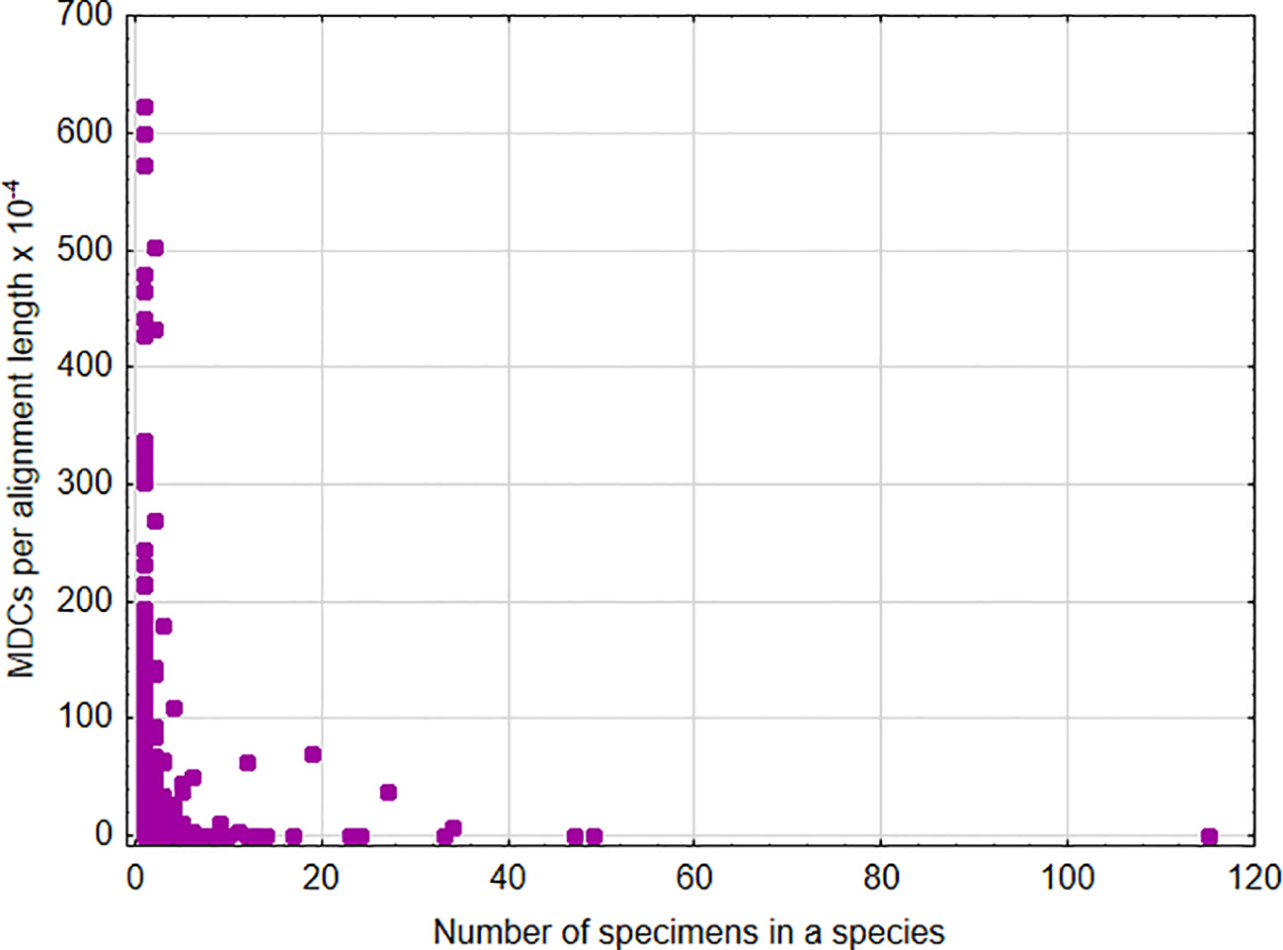
Number of MDCs observed in a species normalized by the length of alignment versus number of analyzed specimens in a species.

A significant positive association was found between the sum of MDCs and the number of species in the genus [parameter = 0.028 ± 0.0002(SEM), Wald stat. = 18036.572, p < 0.001; model: log-likelihood function = -168023.665, *χ^2 = ^* 15872.17, p < 0.00001) (**Figure 1**). This indicates that, in general, an increase in the number of species analyzed within a genus results in the ability to define more MDCs. In turn, a significant negative relationship was found between the cumulative number of MDCs normalized to the alignment length and the number of individuals in the species [parameter = -0.244 ± 0.007(SEM), Wald stat. = 1074.946, p < 0.001; model: log-likelihood function = -29290.708, *χ^2 = ^* 2166.708, p < 0.00001) (**Figure 2**). This implies that a higher number of individuals studied within a genus leads to a reduced capacity to define MDCs.

**Figure 3:**
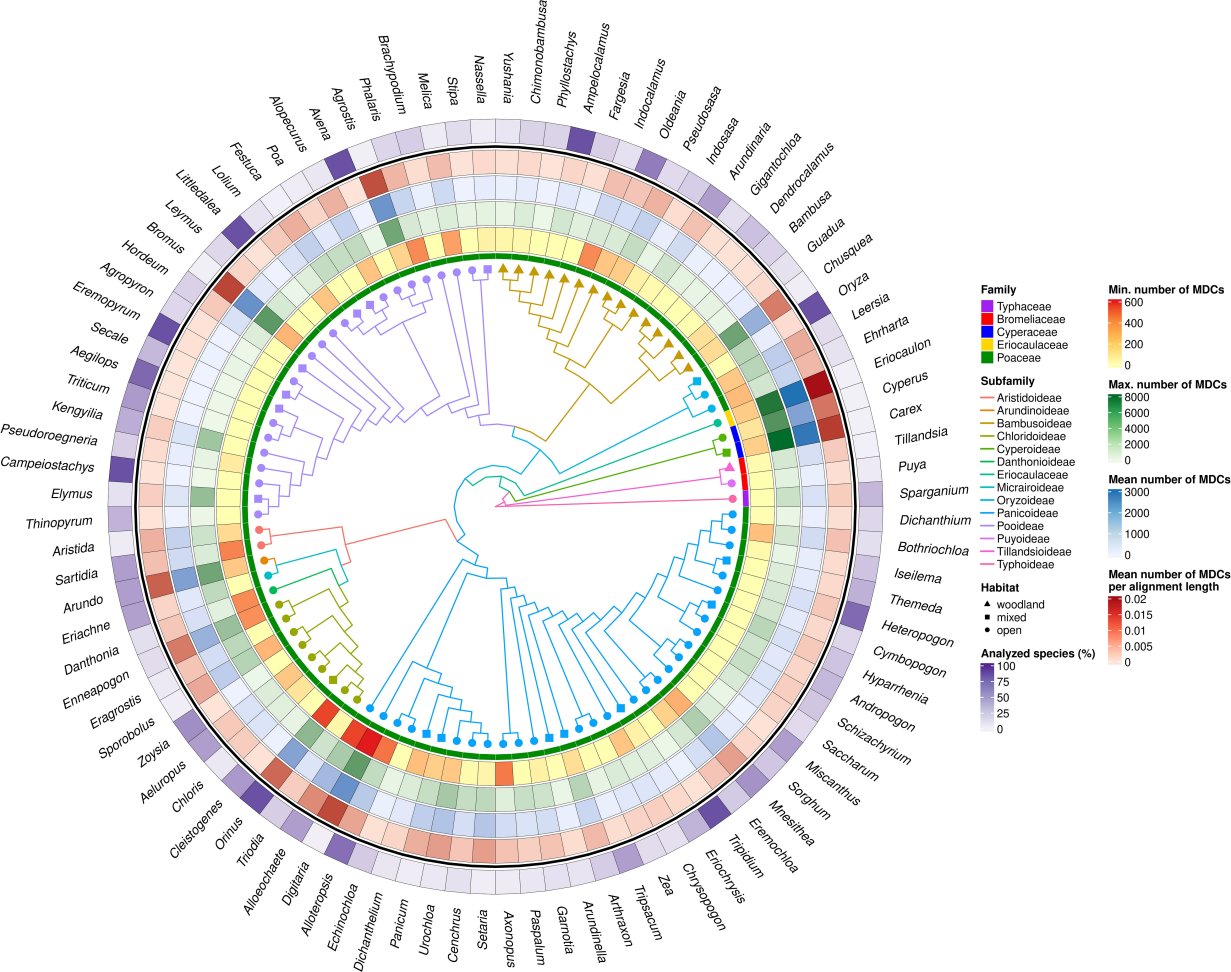
MDCs distribution and phylogenetic relations within Poales order. The heatmaps from most outer describe: the percentage of all species included in the genera MDCs analysis; mean number MDCs per alignment length; mean number of MDCs; maximal values of MDCs; minimal value of MCDs for each genus; and Poaceae family membership. The branch color and node shape of phylogenetic tree depict subfamily belonging and plant habitat, respectively.

The number of species studied within individual genera in some cases was highly representative, reaching up to 100% of species distinguished within the genus (*Ampelocalamus, Avena, Campeiostachys, Eremopyrum, Littledalea, Orinus, Oryza, Tripidium*), although, in the 73 analyzed genera, the availability of data allowed us to analyze less than half of species described (**Figure 3**; **Supplementary Table 2**). However, the representativeness of the analyzed data set in the individual genera does not coincide with their variability, expressed by the number of MDCs (**Figure 3**).

**Figure 4:**
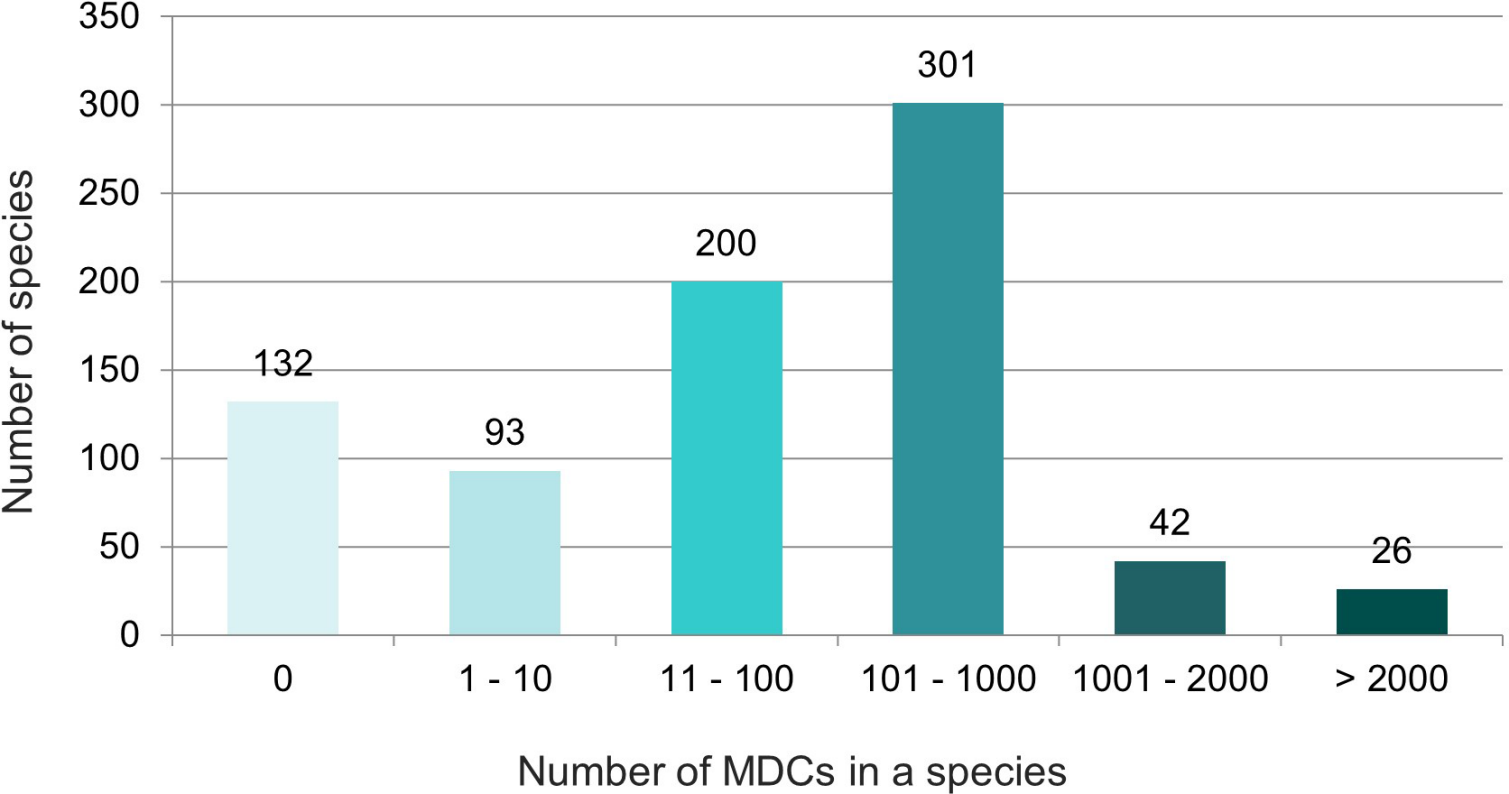
Diversity of the studied species in terms of the number of MDCs characterizing them. The numbers in the chart indicate the number of species. The colors correspond to the number of MDCs in the species.

**Figure 5:**
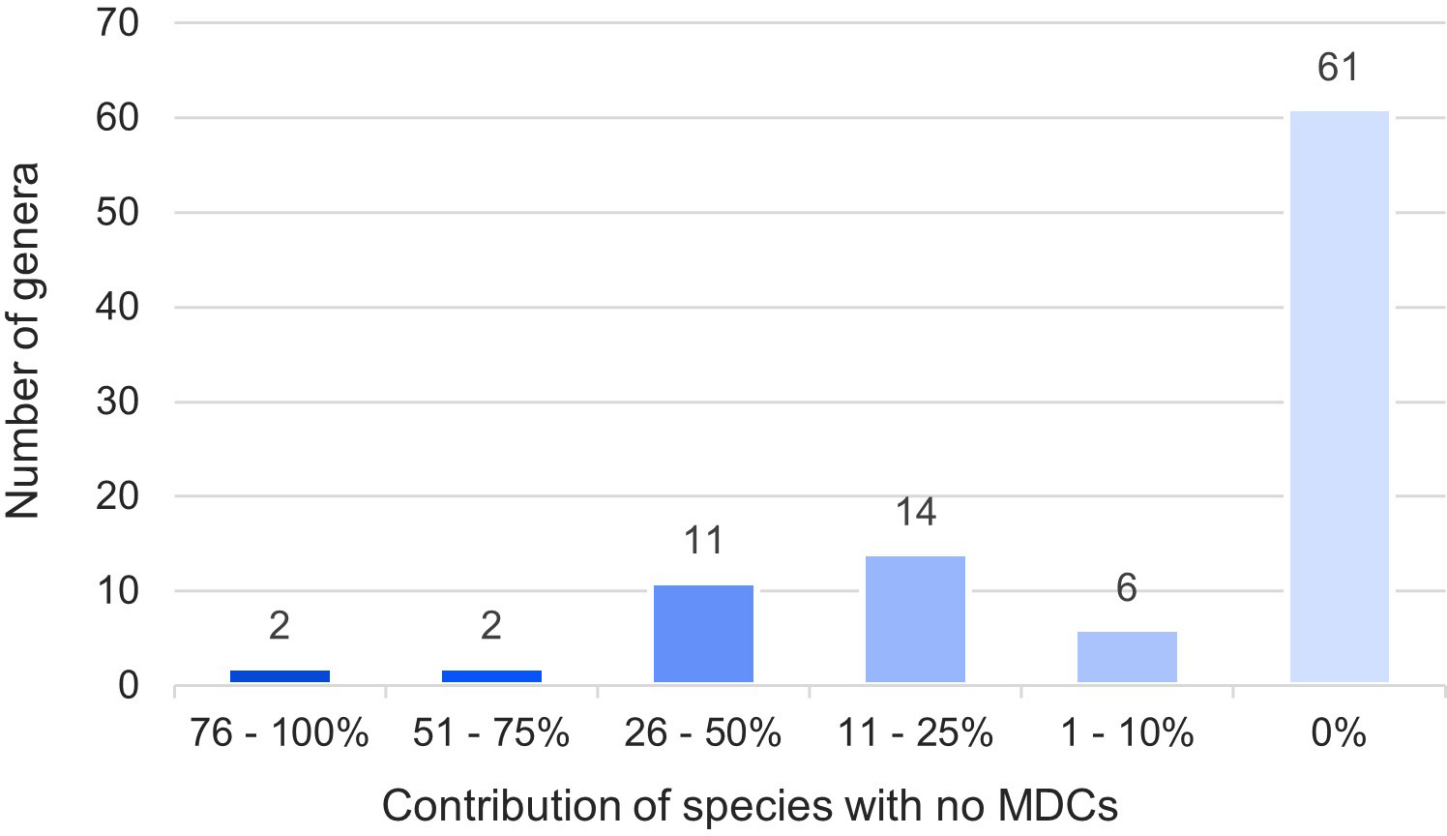
Share of species in a genus impossible to identify. The numbers in the chart indicate the number of types in a given range. The colors correspond to the percentage of species with no MDCs in each genera.

The obtained results showed that in nearly half (46%) of the 794 species over 100 species-specific MDCs were observed (**Figure 4**). On the other hand 93 species (12%) had only from one to ten MDCs in the whole chloroplast genome. Moreover, as many as 132 species (17%) did not have any MDC, therefore they couldn’t be identified on the basis of differences in the complete cpDNA sequence (**Figure 4**). The analysis of the observed number of MDCs in individual genera showed that the studied genera turned out to be very diverse in terms of the share of species in which not a single MDC was defined. (**Figure 5**). In 61 out of 96 analyzed genera, MDCs were observed in each of the studied species. The share of the remaining 35 species without private SNPs ranged from 5% (*Eragrostis*) to 91% (*Triticum*) or even 100% (*Secale*) (**Supplementary Table 2**). It is worth noting that in the case of 15 genera, the share of species impossible to identify exceeded 25% (**Figure 5**). In terms of percentage of species with no MDCs in a genus, *Stipa* ranked among the 13 genera most difficult to identify (**Supplementary Table 2**).

Analyzing the distribution of genera with no MDCs on the phylogenetic tree, it can be seen that some of them with an exceptionally high share of species without MDCs: *Secale*, *Triticum*, *Aegilops*, *Campeiostachys*, *Thinopyrum*, *Eremopyrum*, *Elymus*, *Kengyilia* belong to the phylogenetically youngest part of the clade formed by the subfamily Pooideae (**Figure 3**). Also the genera *Miscanthus*, *Saccharum* and *Sorghum* are closely related and form a single clade within the subfamily Panicoideae. In addition, in its sister clade, there are also present genera in which some species do not have MDCs, namely: *Schizachyrium*, *Andropogon*, *Hyparrhenia*, *Heteropogon* and *Themeda*. A similar picture can be seen in the Bambusoideae for *Gigantochloa*, *Dendrocalamus* and *Bambusa*. The other genera with no MDCs detected are found in different parts of the tree, without a clear relationship with phylogeny (**Figure 3**).

In turn, the studied genera characterized by high interspecific diversity, and therefore with high mean number of MDCs, but above all a high minimum MDCs number, belong to the Cyperaceae and Eriocaulaceae families, but are also observed in the basal part of the Panicoideae clade (*Alloeochaete*, *Digitaria*, *Alloteropsis*). The remaining cases of genera characterized by high MDCs level are located in different parts of the phylogenetic tree.

### 3.2 Mean pairwise genetic identity versus MDCs number

**Figure 6:**
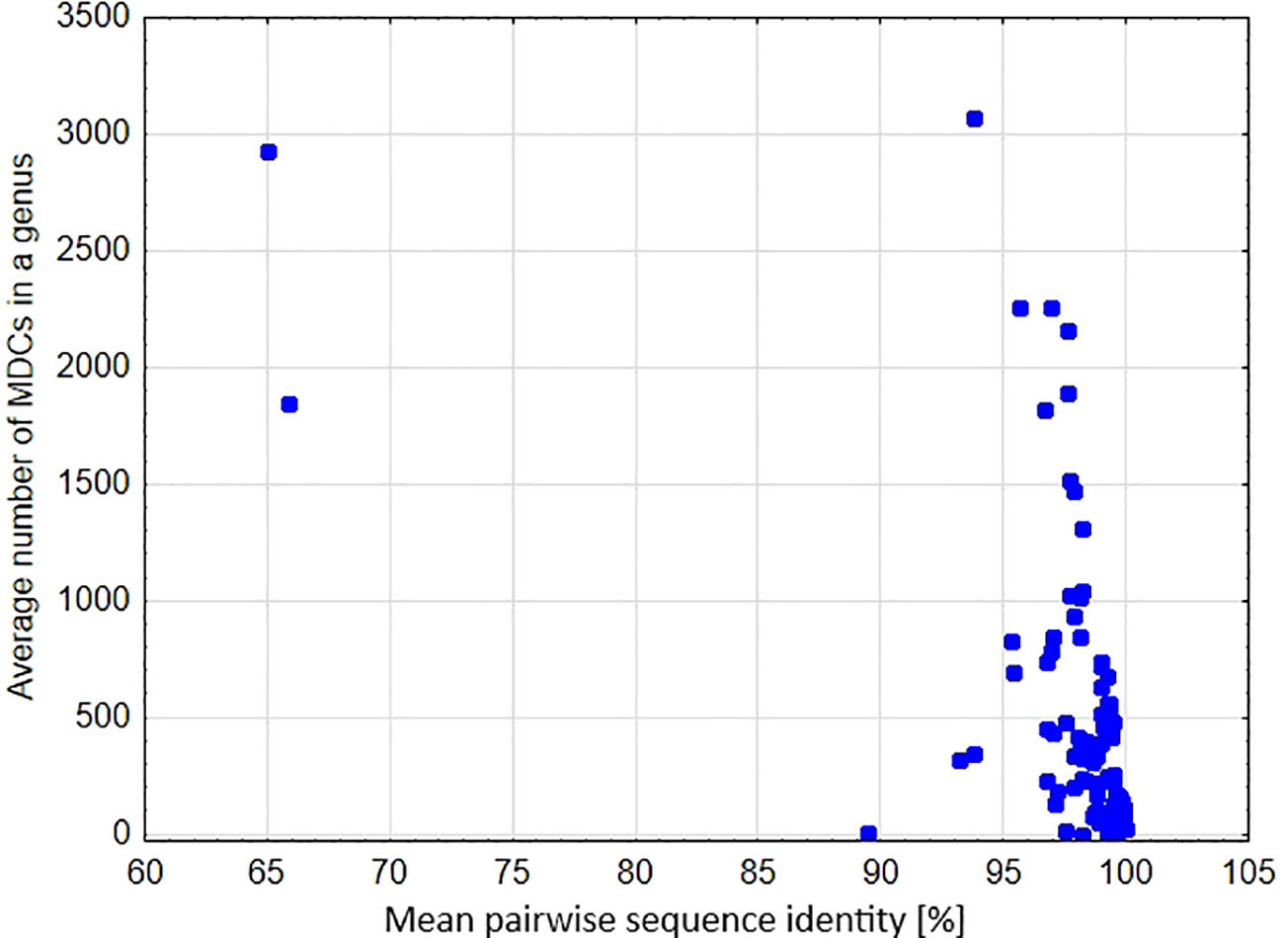
Mean pairwise sequence identity in a genus versus the average number of MDCs calculated for the genus.

**Figure 7:**
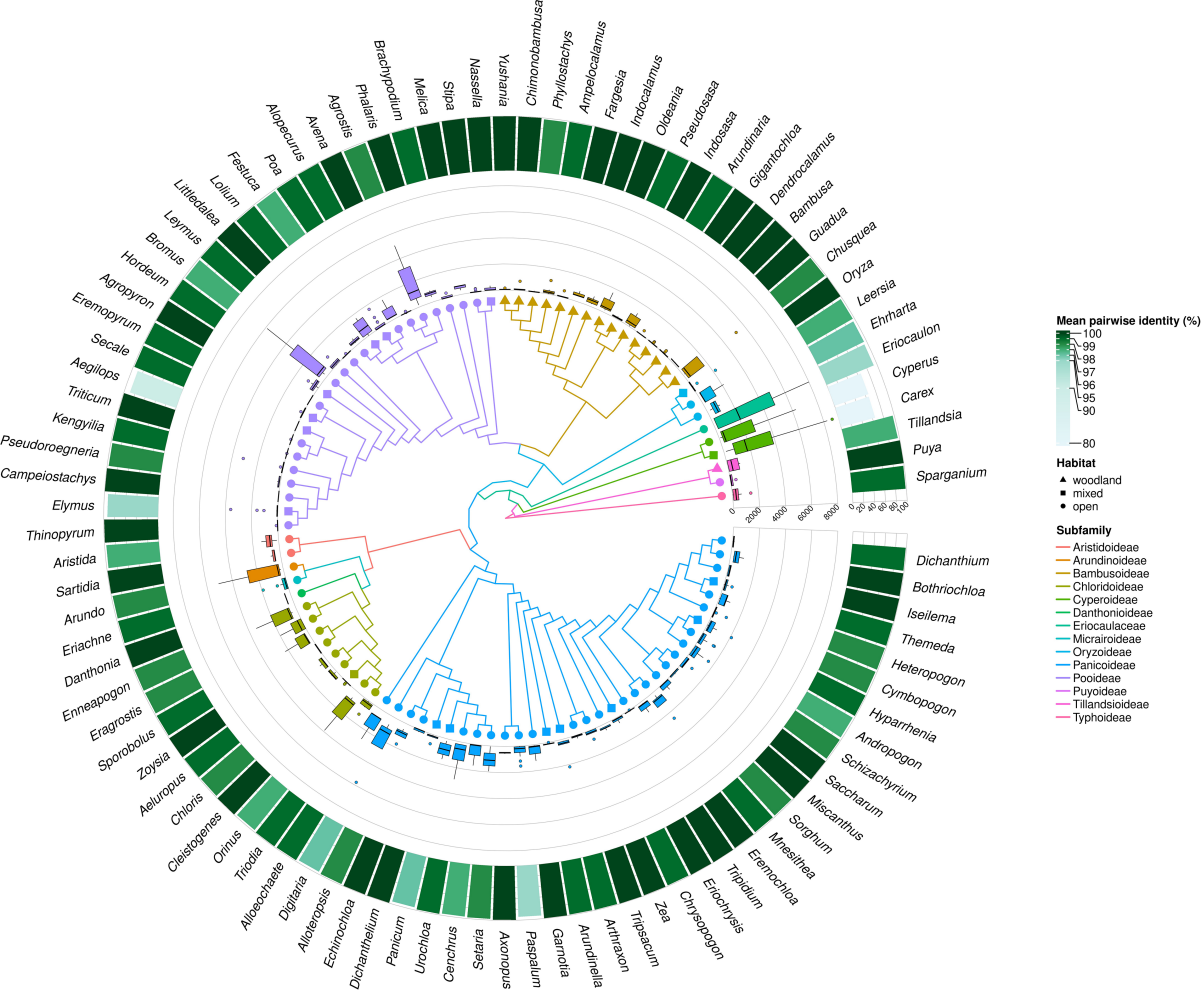
Mean pairwise identity within Poales genera distributed on phylogenetic relationship. The heatmap reveals the percentage of mean pairwise identity calculated for each pair of species within the genus. Boxplot analysis comparing the number of MCDS for species. The boxplot shows the median, first and third quartiles (lower and upper hinges), largest and smallest value (upper and lower whisker). The tree colors describe subfamily membership.

A significant negative relationship was found between the average number of MDCs in the genus and % pairwise identity [parameter = -0.055 ± 0.0004(SEM), Wald stat. = 16454.907, p < 0.00001; Model: log-likelihood function = -29127.302, χ^2 = ^10773.301, p < 0.00001) (**Figure 6**). This result clearly shows that genetic distance calculated as a percentage of mean pairwise sequence variability within a genus largely goes together with the effectiveness of molecular identification of species in a genus. This is also perfectly visible in the example of the genera *Carex*, *Cyperus* and *Eriocaulon*, where high variability of the aligned sequences coincide with high mean and maximum values of MDCs in a genus (**Figure 7**). In turn, there are cases of the opposite situation, as in *Aegilops*, *Elymus* and *Paspalum*, where the high genetic differentiation of sequences does not correspond with identification efficiency. On the other hand, some genera characterized by high sequence similarity: *Phalaris* (99.2%), *Axonopus* (99.3%), *Danthonia* (99.4%) had high minimal an mean MDCs numbers (**Figures 3**, **5**, **7**), thanks to which molecular identification of their species was not a challenge. A surprising situation was also observed in three genera from the subfamily Bambusoideae: *Guadua*, *Indosasa* and *Yushania*, where despite 99.9% sequence identity, the success of species identification in each of these genera was 100%, moreover, the number of MDCs for the species was in the range of 107-132, 46-216 and 32-149, respectively.

### 3.3 Types of mutations generating MDCs

Among species-specific mutations generating MDCs in barcode analysis there were substitutions, single and multi-nucleotide insertions or deletions. Moreover, when comparing query species to other representatives of the genus, a situation was also observed when in query species in a given alignment position there was for example nucleotide A, while in other species there was C, G or T or a deletion in this place, so this type of MDCs we defined as mixed variation. The analysis of the type of MDCs observed in the studied data set showed that in most species (65%) the dominant source of MDCs were indel and mixed variations, in 17% of species these were substitutions, while in 18% of cases their share was equal (**Supplementary Table 3**). The predominance of MDCs of substitution type generally applies to a few species in the genus, however, in the case of *Sparganium*, *Ehrharta* and *Leersia*, their share was predominant in 67% of species of the genus, and in *Puya* and *Bambusa* in 50% of species. In the Poaceae family, the genus *Stipa* stands out in this respect, in which substitutions prevailed in 29% of the analyzed species.

The analysis of the number of MDCs generated by different types of mutations showed that 27.1% of them are in substitution type while as much as 68.1% of observed MDCs in the data set came from multi-nucleotide indels or mixed variations. Detailed data on the contribution of each of the types of mutations defined as MDCs for analyzed species are provided in **Supplementary Table 3**.

### 3.4 Type of habitat

**Figure 8:**
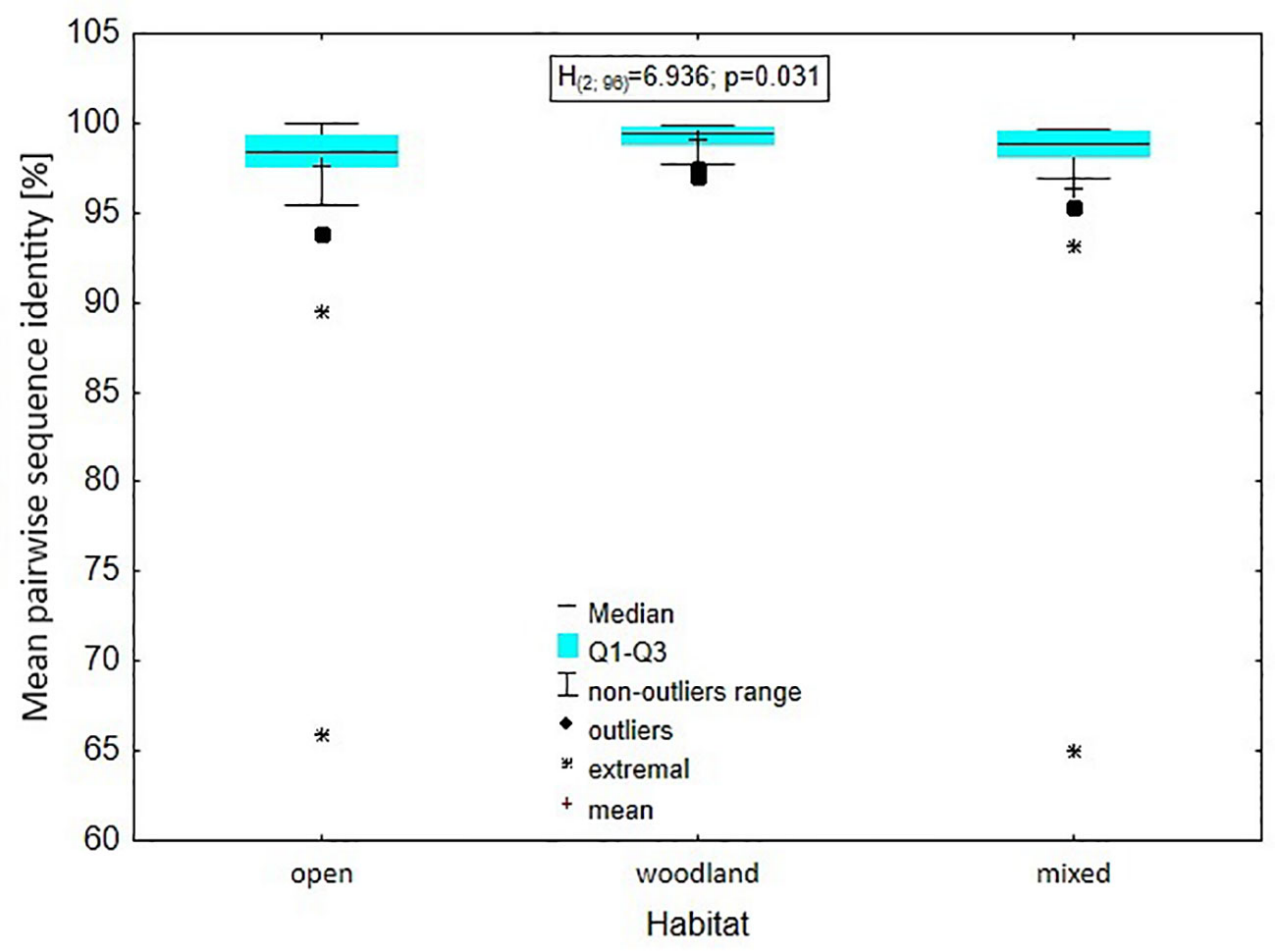
Level of genus variability in relation to the type of habitat: open, closed or mixed, occupied by species representing a given genus.

The analysis of the level of genus variability in relation to the type of habitat: open, closed or mixed, occupied by species representing a given genus showed that there was a significant difference in mean pairwise identity between the types of environments (Kruskal-Wallis test: H(2, N = 96) = 6.936; p = 0.031), with the difference relating to the mean pairwise identity between the open and woodland environments. A higher percentage was found in the woodland environment (Dunn test: z = 2.613, p = 0.027). Between mixed and woodland this difference was within the inference error (Dunn test: z = 1.911, p = 0.168); the situation was similar when testing the differentiation between open vs. mixed environments (Dunn test: z = 0.242, p = 1.000) (**Figure 8**).

## 4 Discussion

### 4.1 Efficacy of species identification within Poales

The results show that a very low level of genetic diversity between plastomes is not uncommon in Poales, since in 18 out of 96 examined genera mean pairwise sequence identity exceeded 99.5%. Furthermore, many of the species studied are impossible to distinguish even when analyzing the entire chloroplast genome due to the lack of species-specific mutations. A particularly large number of cases where super-barcoding failed to discriminate species have been observed in the Poaceae family. Representatives of the remaining families included in this study were generally characterized by high variability of chloroplast genomes, on the basis of which the molecular identification of species was successfully carried out. Although in the genus *Puya* from the family Bromeliaceae and in *Sparganium* from the Typhaceae, the identification success was also not complete.

Unfortunately, application of super-barcodes to identify species from Poales is not yet very common, which hinders a broader discussion on interspecific variability of this group of plants. However, entire chloroplast genome sequences are being used to reconstruct evolutionary history at the genus level. So far, this type of research has been carried out for the *Bambusa* genus (Wysocki et al., 2015), *Festuca* (Hand et al., 2013; Qiu et al., 2019), *Lolium* (Hand et al., 2013), and *Stipa* (Krawczyk et al., 2018). Data on the variability of chloroplast genomes within these genera correspond well with the results of our study reporting similar values of genetic diversity.

### 4.2 Number of MDCs against the background of phylogenetic relationships

The result of phylogenetic reconstruction inferred in our study is only fragmentary, due to the availability of data for analysis, nevertheless, the presented phylogenetic tree is well-resolved and congruent in topology with recent phylogenetic studies on Poales (Saarela et al., 2015; Soreng et al., 2017; Gallaher et al., 2022; Wu et al., 2022). Our study shows that the level of genetic diversity and number of species-specific mutations are extremely variable within different lineages of Poales. There may be many reasons for this phenomenon as the mechanism of plastome evolution is very complex, including: gene losses, gene duplications, gene rearrangements, gene transfers between organelles, horizontal gene transfer, repeat sequences and point mutations (Bennetzen, 2007; Jansen and Ruhlman, 2012; Xu et al., 2015; Wu et al., 2022).

Within Poales, the most dynamically evolving group was the evolutionary branch leading to the grass family. The result of the accelerated rate of evolution was that the common ancestor of the Poaceae family lost from plastome introns in two genes: *clp*P and *rpo*C1 and three coding regions: *acc*D, *ycf*1 and *ycf*2 (Guisinger et al., 2010; Xu et al., 2015; Liu et al., 2020). The lack of these genome fragments, especially the *ycf*1 gene, which is particularly useful in molecular identification of species of land plants, being a source of numerous species-specific mutations (Dong et al., 2015), may be one of the reasons for the generally low interspecific diversity of plastomes observed in the grass family.

The mutation rate may also be influenced by selective pressure, which is variated in individual evolutionary lines of Poales (Guisinger et al., 2010; Piot et al., 2018; Krawczyk et al., 2022). Literature data also suggest that accelerated rates of both genome rearrangements and nucleotide substitutions in the chloroplast genome may possibly be caused by aberrant DNA repair mechanisms which result from mutations in nuclear-encoded DNA repair and/or replication genes (Jansen et al., 2007; Guisinger et al., 2010; Guisinger et al., 2011).

### 4.3 Super-barcoding in Poales versus other groups of plants

So far, there are also not many publications reporting the number of species-specific mutations in complete plastome sequences. Although the super-barcoding method is gaining in popularity as well as usage of molecular identification of species by defining MDCs, these two methodological approaches rarely go hand in hand in plant research. Regarding plastomes, we have scant data on selected liverworts, which show that between two species representing the genus *Marchantia* (*M. polymorpha* and *M. palaceae*) 4,076 MDCs have been defined, while in the genus *Conocephalum* (*C. conicum* and *C. salebrosum*) 5,878 were observed (Sawicki et al., 2020). In turn, in the genus *Pellia* and *Apopellia* the number of detected plastid MDCs varied from 0 to 4,717 (Paukszto et al., 2023), including 159-1,369 MDCs identified for cryptic species within the genus *Apopellia*. Comparison of these results with those obtained for Poales, where the highest average value of MDCs for the genus was 3,071 (in *Eriocaulon*), while the highest observed value for the species was over 32 k bp (in *Carex*), shows that the number of MDCs in Poales is extremely volatile, albeit at a fairly low level on average.

In the case of vascular plants, we can refer to data on the comparison of three species representing the genus *Pulsatilla*, where species-specific mutations ranged from 56 to 432 (Szczecińska and Sawicki, 2015). In the study on *Pulsatilla*, for each species, the number of specific substitutions was at least twice the number of species-specific indels. In Poales we observed almost three-fold advantage of indel and mixed MDCs over substitutions, as well as the overwhelming advantage of long indels over single ones, which is a good reflection of the rapid evolutionary changes that shaped the variability of Poales representatives. Long indels, which generate most of observed MDCs, have a particularly large impact on the efficiency of species identification within this group of plants.

### 4.4 Reasons for the low effectiveness of super-barcoding

Observations of low success of plant species identification with super-barcoding in some cases can be explained by the high level of genetic sequence similarity within a genus, however the variability of plastomes is not always consistent with their diagnostic capability in molecular fingerprinting. This means that despite a large share of variable sites found within a plastome sequence, it may be characterized by a low share of private mutation sites, and thus - poor efficiency of species identification. Therefore, variability does not always prove the effectiveness of distinguishing taxa, because it does not have to be species-specific.

When looking for the reasons for the low efficiency of molecular identification of species by DNA barcoding in selected groups among Poaceae, it should be noted that many species in this group evolved by polyploidization, since most grass species examined against ploidy are polyploids (Rice et al., 2015). The phenomenon of polyploidization can lead to problems related to the identification of species by DNA barcoding based on the plastid genome. This is due to the fact that the newly formed autopolyploids are characterized by an identical chloroplast genome as the parent species. Differences within the plastid genome may accumulate over time, however, at least in the early generations following the polyploidization process, the aforementioned difficulties in species identification will occur. Recently formed allopolyploids will, in turn, have the chloroplast genome of only one of the species, which makes it impossible to distinguish them from the taxon from which the given plastid genome originates using the DNA barcoding method (Cowan and Fay, 2012). Unfortunately, for the vast majority of species studied in this study, the literature does not provide the number of chromosomes on the basis of which the contribution of polyploid species to the genus could be assessed. Thus, we do not have a complete picture of the share of polyploid species in individual genera. However, based on the data available in The Chromosome Counts Database (CCDB) (Rice et al., 2015), it can be concluded that the genera in which the success of species identification is particularly low include species with varying degrees of polyploidy. For example, the genus *Triticum* includes di-, tri-, tetra- and hexaploid species, the genera *Aegilops* and *Avena*: di-, tetra-, hexaploids, and *Oryza* di-, tetra-, hexa- and octoploids.

Another important mechanism of species evolution that reduces the effectiveness of species identification based on the comparison of chloroplast sequences is the phenomenon of hybrid origin of species and introgression across species barriers (Soltis and Soltis, 1991; Li et al., 2015). There are numerous known cases of both ancient and recent hybridizations and subsequent genomic introgressions between grass species, to name just a few genera analyzed in the study: *Lolium* (Jenkins, 1986), *Phyllostachys* (Lin et al., 2010), *Stipa* (Nobis et al., 2019; Baiakhmetov et al., 2020), *Urochloa* (Tomaszewska et al., 2023), *Aegilops*, *Secale*, *Triticum* and *Sorghum* (Kole, 2011).

An important factor hindering species identification based on the plastome may be a phenomenon of chloroplast capture through interspecies hybridization, which yields a plant with a new genetic combination of nuclear and chloroplast genomes (Rieseberg and Soltis, 1991). Hybridization phenomena and probable plastome capture have probably occurred many times in Poales, such as in the Triticeae between *Thinopyrum* and *Pseudoroegneria* or *Aegilops* and *Triticum* (Petersen et al., 2006; Bernhardt et al., 2017), as well as between species within the genus *Stipa* (Nobis et al., 2019) or *Zea* (Morales-Briones et al., 2018; Wu et al., 2022). Another reason for the whole plastome lateral transfer between species may be asexual modes like natural grafting, which may occur when two plant roots contact each other (Stegemann and Bock, 2009; Stegemann et al., 2012), or in the case of transfer using a parasitic vector (Stegemann et al., 2012; Gao et al., 2014).

### 4.5 The influence of the occupied habitat on interspecies variability

The assessment of plastome variation and species-specific diversity concerning the habitats in which the studied species currently reside revealed the significant impact of this ecological factor on the development of interspecies diversity within Poales. In the genera which representatives occupy only open habitats, the diversity is significantly greater than in the case of genera occurring in woodlands and mixed habitats, which is probably related to the influence of the habitat type on the biology of pollination and seed dispersal. The influence of the occupied habitat on shaping the variability of Pooideae at earlier stages of the development of this phylogenetic line was previously demonstrated by Zhang and colleagues (Zhang et al., 2022). According to their study the transition from closed to open habitat which occurred at the turn of the Eocene and Oligocene coincided with an upshift of diversification rate in core Pooideae. The results showed that members of three Pooideae lineages transitioned parallelly to open habitats: the most recent common ancestor (MRCA) of supertribe Stipodae, the MRCA of the core Pooideae, and the MRCA of supertribe Nardodae. This event also coincided with a cooling of the climate, and a massive increase in gene and genomic duplications (Zhang et al., 2022). This indicated physiological and morphological innovations which subsequently contributed to plant adaptation to new or stressful environments. The combination of internal genetic events and external environmental stimuli resulted in the radiation of Pooideae into multiple successful lineages with wide expansion to new territories in temperate regions (Soltis and Soltis, 2016).

### 4.6 Non-biological factors affecting molecular identification of species

In addition to biological factors, the effectiveness and reliability of the DNA barcoding method are influenced by two issues that should be considered. The first is the level of knowledge of a given genus and the compliance of its division into species adequate to genetic variability. The taxonomic division within the genus is often contentious, and dependent on the adopted definition of the biological species. The status of taxa at the level of species and subspecies changes dynamically, as well as the affiliation of species to particular genera. This is perfectly illustrated by a study of the genus *Cephalotaxus*, where the effectiveness of species identification using the super-barcoding method was compared depending on the taxonomic division used (Wang et al., 2022). The second issue is the presence in GenBank of reference sequences that have been deposited for mislabeled taxa. The scale of this phenomenon is unknown, although to some extent it affects the results of comparative analyses, probably also in the case of these studies. The problem of correct species identification on the example of *Oryza* genus and mislabeled plant material from seed banks was pointed out by Zhang and colleagues (Zhang et al., 2021). Situations when we analyze sequences incorrectly assigned to a given species result in the fact that we try to find species-specific mutations for genetically distant sequences, which leads to an artificial reduction in the number of defined MDCs. However, in order to capture such situations, each sequence that differs significantly from other representatives of the species should be verified at the source, and thus reach the material herbarium. In turn, the elimination of outlier sequences from the dataset does not seem legitimate either, unless we are sure that it is a mislabeled species, because there is a risk that in turn we could artificially reduce genetic diversity in some cases and increase the number of species-specific characters. This leads to the conclusion that regardless of whether we use the method of species identification based on the phylogenetic tree, on the analysis of genetic distance or on the analysis of MDCs, the basis for the reliability of the DNA barcoding method is the adequate taxonomic order in the studied group and excellent knowledge of the species affiliation of the examined representatives.

## 5 Conclusions

Our study revealed huge diversity in plastome interspecies variability and the number of species-specific mutations within Poales. The efficacy of the molecular species identification method in the analyzed data set generally was associated with the level of plastome genetic diversity, however, cases deviating from this rule have also been reported. It was observed that a particularly large number of species without species-specific mutations belong to the evolutionarily youngest genera within the Pooideae and Panicoideae, although they have also been noted in other parts of the Poales phylogenetic tree. It has also been shown that in Poales the genera whose representatives occur only in open areas are characterized by a higher level of plastome variability than genera occupying forest habitats. Polyploidization and hybridization of species seem to be of particular importance for reducing the effectiveness of super-barcoding, although the scale of this phenomenon is not yet well studied.

## Data availability statement

The original contributions presented in the study are included in the article/**Supplementary Material**. Further inquiries can be directed to the corresponding author.

## Author contributions

KK: Conceptualization, Formal analysis, Investigation, Methodology, Supervision, Writing – original draft. ŁP: Formal analysis, Visualization, Writing – original draft, Methodology. MM: Visualization, Writing – review & editing. JS: Conceptualization, Formal analysis, Investigation, Methodology, Supervision, Visualization, Writing – review & editing.

## Supplementary material

Supplementary Figure 1 The phylogenetic Poales tree built using the Maximum Likelihood method implemented in the IQ-TREE 2 from consensus sequences calculated for the analyzed plant genera. The booststrap values are given above branches. Genus names given in red indicate taxa with the highest share of species with no MDCs discussed in the text.
