# Supplementary figures and images for: The low level of plastome differentiation observed in some lineages of Poales hinders molecular species identification
Source: Front Plant Sci. 2023 Dec 8;14:1275377. doi: 10.3389/fpls.2023.1275377 (PMC10739336; doi:10.3389/fpls.2023.1275377)

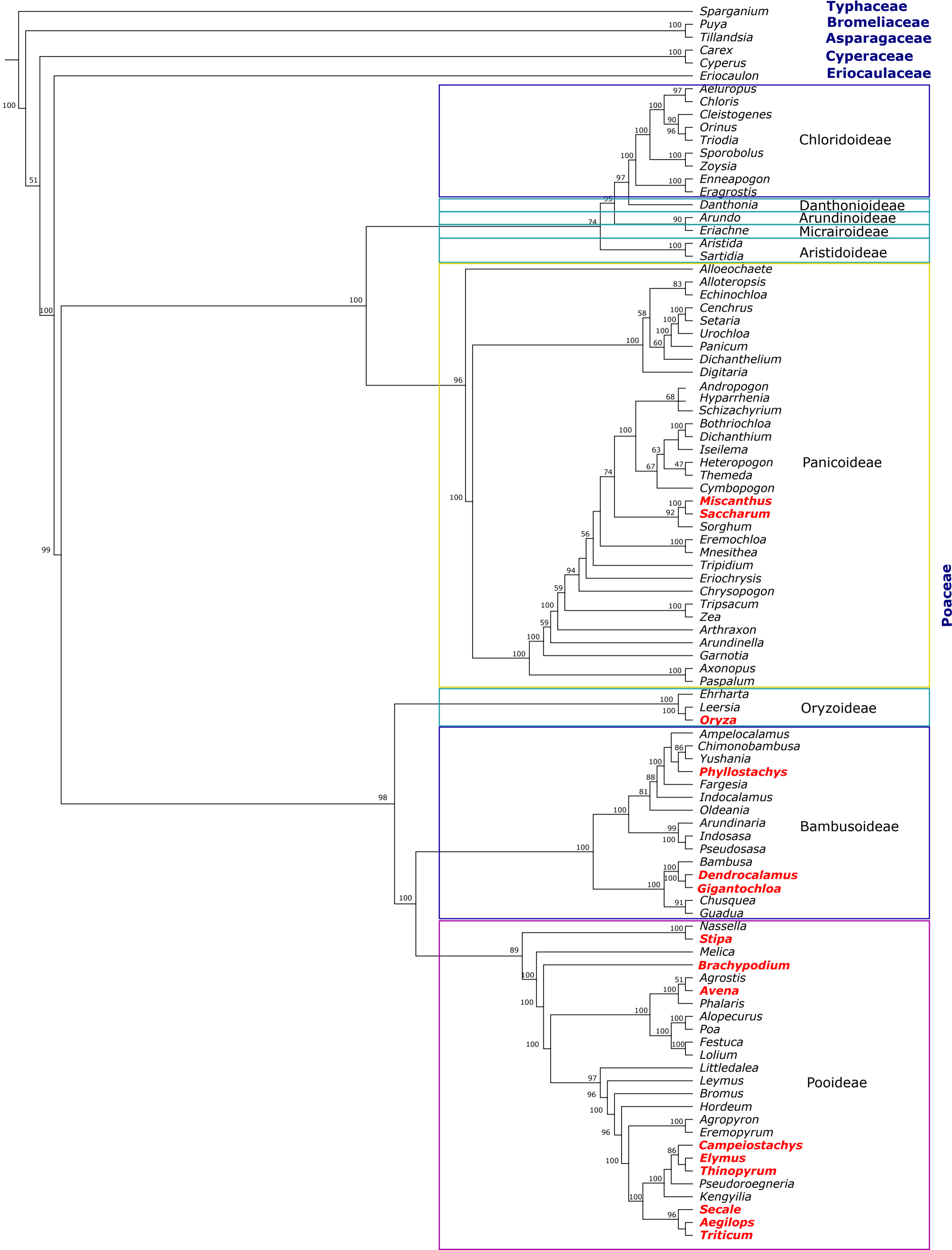

Supplement: Supplementary Figure 1 — The phylogenetic Poales tree built using the Maximum Likelihood method implemented in the IQ-TREE 2 from consensus sequences calculated for the analyzed plant genera. The booststrap values are given above branches. Genus names given in red indicate taxa with the highest share of species with no MDCs discussed in the text. [file DataSheet_1.pdf]
